# Replication of progressive supranuclear palsy genome-wide association study identifies *SLCO1A2* and *DUSP10* as new susceptibility loci

**DOI:** 10.1186/s13024-018-0267-3

**Published:** 2018-07-09

**Authors:** Monica Y. Sanchez-Contreras, Naomi Kouri, Casey N. Cook, Daniel J. Serie, Michael G. Heckman, NiCole A. Finch, Richard J. Caselli, Ryan J. Uitti, Zbigniew K. Wszolek, Neill Graff-Radford, Leonard Petrucelli, Li-San Wang, Gerard D. Schellenberg, Dennis W. Dickson, Rosa Rademakers, Owen A. Ross

**Affiliations:** 1 Department of Neuroscience, Mayo Clinic, 4500 San Pablo Road, Jacksonville, FL 32224 USA; 2 Department of Pathology, University of Washington, Seattle, WA USA; 3 Department of Health Sciences Research, Mayo Clinic, Jacksonville, FL USA; 4 Division of Biomedical Statistics and Informatics, Mayo Clinic, Jacksonville, Florida, USA; 5 Department of Neurology, Mayo Clinic, Scottsdale, AZ USA; 6 Department of Neurology, Mayo Clinic, Jacksonville, FL USA; 7 Penn Neurodegeneration Genomics Center, Department of Pathology and Laboratory Medicine, Perelman School of Medicine, University of Pennsylvania, Philadelphia, PA USA; 8 Department of Clinical Genomics, Mayo Clinic, Jacksonville, FL USA

## Abstract

**Background:**

Progressive supranuclear palsy (PSP) is a parkinsonian neurodegenerative tauopathy affecting brain regions involved in motor function, including the basal ganglia, diencephalon and brainstem. While PSP is largely considered to be a sporadic disorder, cases with suspected familial inheritance have been identified and the common MAPT H1haplotype is a major genetic risk factor. Due to the relatively low prevalence of PSP, large sample sizes can be difficult to achieve, and this has limited the ability to detect true genetic risk factors at the genome-wide statistical threshold for significance in GWAS data. With this in mind, in this study we genotyped the genetic variants that displayed the strongest degree of association with PSP (*P*<1E-4) in the previous GWAS in a new cohort of 533 pathologically-confirmed PSP cases and 1172 controls, and performed a combined analysis with the previous GWAS data.

**Results:**

Our findings validate the known association of loci at MAPT, MOBP, EIF2AK3 and STX6 with risk of PSP, and uncover novel associations with SLCO1A2 (rs11568563) and DUSP10 (rs6687758) variants, both of which were classified as non-significant in the original GWAS.

**Conclusions:**

Resolving the genetic architecture of PSP will provide mechanistic insights and nominate candidate genes and pathways for future therapeutic intervention strategies.

**Electronic supplementary material:**

The online version of this article (10.1186/s13024-018-0267-3) contains supplementary material, which is available to authorized users.

## Background

Progressive supranuclear palsy (PSP) is a Parkinsonian neurodegenerative disorder that presents with predominant 4R tauopathy in basal ganglia, diencephalon and brainstem with associated neuronal loss and fibrillary gliosis [1, 2]. Although PSP is largely considered to be a sporadic disorder, cases with suspected familial inheritance and cases carrying pathogenic mutations have been reported; e.g. mutations in the *MAPT* gene, encoding the tau protein, have been associated with PSP phenotypes [3]. In addition, the common *MAPT* H1 haplotype is established as the major genetic risk locus for PSP [4–6].

The only unbiased genome-wide association study (GWAS) to date in PSP was performed in a total cohort of 2165 PSP patients and 6807 controls [7]. The discovery-replication design confirmed the *MAPT* locus as the most strongly associated genetic risk factor (OR = 5.46; *P* = 1.5E-116). The study also identified three novel loci associated with disease susceptibility *MOBP* (OR = 0.72; *P* = 1.0E-16), *STX6* (OR = 0.79; *P*-value = 2.3E-10) and *EIF2AK3* (OR = 0.75; P-value = 3.2E-13). Follow-up studies have attempted to draw more specific associations of these variants with the risk of PSP. Sequencing of the coding regions of the GWAS implicated genes in 84 PSP cases was mainly negative with the exception of a rare, predicted damaging *STX6* p.C236G mutation that remains of unknown relevance [8] and the association of the *EIF2AK3* haplotype B, known to be in LD with rs7571971, with the risk of PSP [9].

Due to the relatively low prevalence of PSP, large sample sizes can be difficult to achieve, and this can result in a GWAS having less than desirable power to detect biologically meaningful associations at the genome-wide statistical significance threshold. Thus, maximizing sample size (the number of PSP patients in particular) is imperative in order to reduce the likelihood of obtaining false-negative findings for true genetic risk factors for PSP, and meta-analytic studies are an effective way to accomplish this. Therefore, in the current study we have included a new cohort of 533 pathologically-confirmed cases and 1172 controls, genotyped the top variants identified in the original GWAS (*P* < 1E-4), and performed a combined analysis with the original GWAS data in order to attempt to confirm the previously reported genes and also identify additional candidates.

## Methods

### Study sample

This study included 533 pathology-confirmed PSP patients, all confirmed negative for *MAPT* mutations. These patients have donated their brains to the Mayo Clinic brain bank for neurodegenerative disorders. It should be noted the Mayo Clinic brain bank receives cases from across the United States and thus may house a small number of cases that overlap with longitudinal clinical studies. Neuropathologic diagnosis was rendered by a single neuropathologist (DWD) and followed published criteria for PSP [10]. Clinical and demographic information was collected from available medical records. Study controls were approximate age- (±5 years) and gender-matched 1172 clinical volunteers (~ 1:2 case-control ratio) who were observed not to have a neurodegenerative or neurological condition within the Department of Neurology, Mayo Clinic. Additionally, the control population included samples from 106 pathologic-defined control subjects that did not have significant neuropathology suggestive of disease and that have a Braak stage < 3. All PSP patients and controls were unrelated and of European ancestry which was determined by extracting the self-reported ethnicity from medical records. A thorough review of the new PSP cases was performed to manually include only cases that were not part of the original PSP GWAS. Study subjects were recruited through protocols approved by the Mayo Clinic institutional review board.

### SNP genotyping

Selection of follow-up SNPs was performed from the publicly available PSP GWAS results (https://www.niagads.org/datasets/ng00045). The selection was made based on these criteria: 1. Include only the European ancestry analysis results because as previously mentioned the new pathology-confirmed PSP cohort is exclusively composed of Caucasian samples, 2. Include all unique loci with an arbitrary cut-off of *P* < 10^− 4^ in the joint analysis (*N* = 31), and 3. Since our new PSP cohort is all pathology-confirmed cases we considered it to be comparable to the stage 1 GWAS cohort and therefore reasonable to include additional unique loci with *P* > 10^− 4^ in the joint analysis but with P < 10^− 4^ in the stage 1 analysis (*N* = 9). To select only unique chromosomal regions, the list of variants was carefully examined to identify genomic regions by grouping SNPs closely located and in linkage disequilibrium (LD). LD data was obtained from HaploReg v4 (http://archive.broadinstitute.org/mammals/haploreg/haploreg.php) and the 1000 Genomes (Utah Residents (CEPH) with Northern and Western Ancestry). When a region was represented by more than one SNP, we selected the one with highest association score. For *MAPT*, we also included the *MAPT* rs242557 to account for the association of PSP risk and ‘H1c’ haplotype.

In total, 37 SNPs representing the 36 different loci were genotyped in the new pathology-confirmed PSP cohort and controls (Table 1). To prevent genotyping errors, we selected one more SNP that was in high LD with each of the GWAS SNPs (proxy) and genotyped them in parallel on the MassArray genotyping platform. The rs242557 variant tagging the *MAPT* H1c subhaplotype was added to the study and it is routinely genotyped by TaqMan assay in our laboratory. rs11532787 variant did not fit the iPlex design and it was genotyped by Sanger sequencing. MassArray assays were run using the iPlex Gold chemistry (Agena Bioscience, San Diego, CA).

**Table 1:** Demographic characteristics of the new PSP cohort

Age (at last visit or at Death)	All	Females	Males
PSP			
Mean (± SD)	75 (± 7.6)	76 (± 7.7)	74 (± 7.4)
Range (minimum-maximum)	42 (54–96)	36.8 (57–94)	42 (54–96)
Count	533	240	293
Controls (All)			
Mean (± SD)	77 (± 8.7)	77 (± 8.7)	76 (± 8.7)
Range (minimum-maximum)	71 (27–98)	71 (27–98)	64 (33–97)
Count	1172	557	615
Controls (Clinical)			
Age (at last visit)			
Mean (± SD)	76 (± 8)	76 (± 7.5)	76 (± 8.4)
Range (minimum-maximum)	43 (54–97)	39 (57–96)	43 (54–97)
Count	1066	497	569
Controls (pathology)			
Age (at death)			
Mean (± SD)	82 (± 12.9)	82 (± 14.7)	83 (± 10.3)
Range (minimum-maximum)	71 (27–98)	71 (27–98)	63 (33–96)
Count	106	60	46

### Statistical analysis

For the analysis of the new PSP patients and controls that were included in the current study, associations between SNPs and risk of PSP were evaluated using logistic regression models that were adjusted for age and sex in PLINK version 1.07. To be consistent with the original GWAS, the presented odds ratios (ORs) and 95% confidence intervals (CIs) are based on the additive effect of an additional major allele (and only the population of controls are considered in determining the major allele). We used the excessive missingness as a genotype quality control excluded individuals that were missing 80% or more of the 37 genotyped SNPs, call rates for all SNPs were > 95%, and SNPs with HWE *P*-values<.001 in controls were excluded from further analyses. A meta-analysis was performed using fixed-effects models in METAL [11] by combining the genotypes of the selected 37 SNPs of our new data of 533 PSP patients and 1172 controls and genotypes of the sample previously included in the PSP GWAS [7]. Only samples of Caucasian European ancestry were included and METAL used the combined Stage1 and Stage 2 values for meta-analysis. The standard error approach was employed, weighting individual project beta coefficients by the standard error. The threshold for genome-wide significance in our meta-analysis was considered to be *P* ≤ 5 × 10^− 8^; all statistical tests were two-sided.

### Functional annotation

A possible cis-effect of the significant SNP as eQTL (expression quantitative locus) was studied by querying the publicly available GTEx, Braineac, Regulome and PhenGen databases for expression and regulatory information associated with the SNP and all the markers with r2 > 0.8 (GWAS signal set) in Haploreg and 1000Genomes. Circular representation of genomic information associated with SNPs was done using the Package “circlize” for R [12].

## Results

**Fig. 1:**
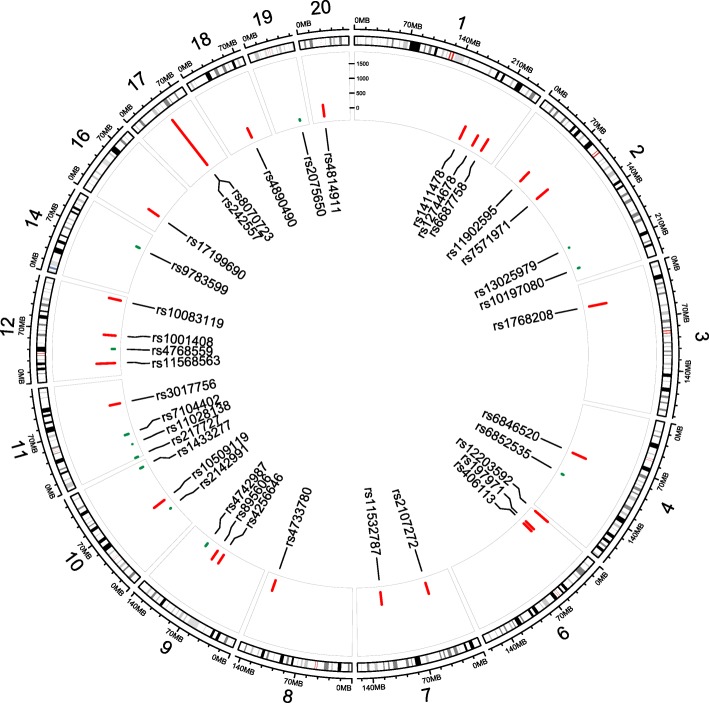
Circos plot summarizing PSP meta-analysis results. The meta-analysis effect of each SNP is plotted in an adjusted scale (meta-analysis effect × 1000). The direction of association is shown with different colors: same direction in both analyses as red lines and opposite direction of association in both analyses as green lines

Ten subjects were excluded for low call rates, leaving 526 cases and 1167 controls. In total, our combined analysis of 37 variants included 934 clinical and 1764 pathology-confirmed PSP (2698 PSP patients in total) and 8019 controls [7]. Characteristics of this new PSP cohort are shown in Table 1. For the 37 genetic variants that were included in this study, associations are shown in Table 2 for the original GWAS, our independent patient-control series from the current study, and the meta-analysis of the original GWAS and our new data. A summary and graphic representation of the results of meta-analysis are presented in (Fig. 1). Eight variants were found to be genome-wide significantly associated with PSP (*P* < 5E-8). The six top significant variants were in *MAPT* (rs8070723 (H1-H2 SNP); and rs242557 conditioned on rs8070723), *MOBP* (rs1768208), *IRF4* (rs12203592), *EIF2AK3* (rs7571971), and *STX6* (rs1411478). Of note, despite displaying a genome-wide significant association in the original GWAS, the association at *IRF4* was not emphasized in that study due to a potential age-related bias. The association with PSP for *IRF4* rs12203592 was also genome-wide significant in our combined analysis however this effect was solely driven by the initial GWAS and our newly genotyped samples did not contribute to the association (OR = 1.09, *P* = 0.40). Thus, age may be influencing the allelic frequency of rs12203592 in the control population and requires further study to resolve the association of this variant with PSP.

**Table 2:** Summary of PSP GWAS and PSP meta-analysis

						GWAS Hoglinger et al. (EUR only)						New Mayo case-control population				Meta-analysis		
						GWAS Stage 1		GWAS Stage 2		Joint Analysis								
SNP ID	chr	GRCh38 location	Gene or nearby gene	Major allele	Minor allele	MAF cases	MAF control	MAF cases	MAF control	OR	P_J_	MAF cases	MAF control	OR (95% CI)	P	OR (95% CI)	P	OR Direction
rs8070723	17	46,003,698	MAPT	A	G	0.051	0.235	0.060	0.232	5.46	1.5E-116	0.049	0.233	5.74 (4.24–7.78)	1.9E-29	5.51 (4.83–6.28)	5.57E-144	++
rs242557	17	45,942,346	MAPT	G	A	0.530	0.348	0.495	0.355	0.51	4.2E-70	0.585	0.403	0.52 (0.45–0.61)	1.2E-18	0.51 (0.48–0.55)	3.78E-85	–
rs1768208	3	39,481,512	MOBP	G	A	0.357	0.286	0.353	0.287	0.72	1.0E-16	0.389	0.264	0.56 (0.48–0.66)	6.6E-13	0.69 (0.64–0.74)	3.48E-26	–
rs12203592	6	396,321	IRF4	C	T	0.164	0.200	0.131	0.198	1.49	6.2E-15	0.159	0.173	1.09 (0.89–1.33)	3.9E-01	1.40 (1.28–1.53)	2.31E-13	++
rs7571971	2	88,595,833	EIF2AK3	C	T	0.314	0.257	0.307	0.248	0.75	3.2E-13	0.320	0.291	0.88 (0.75–1.03)	1.0E-01	0.77 (0.72–0.83)	2.76E-13	–
rs1411478	1	180,993,146	STX6	G	A	0.496	0.418	0.464	0.426	0.79	2.3E-10	0.473	0.410	0.77 (0.66–0.89)	4.4E-04	0.79 (0.74–0.84)	7.17E-13	–
rs11568563	12	21,304,500	SLCO1A2	A	C	0.078	0.053	0.077	0.055	0.69	2.0E-07	0.089	0.053	0.60 (0.45–0.80)	4.2E-04	0.67 (0.59–0.76)	5.26E-10	–
rs6687758	1	221,991,606	DUSP10	A	G	0.229	0.189	0.227	0.191	0.8	2.8E-07	0.230	0.192	0.80 (0.67–0.96)	1.7E-02	0.80 (0.74–0.86)	1.14E-08	–
rs197971	6	23,586,904	LOC105374976	G	A	0.403	0.453	0.410	0.448	1.18	6.7E-06	0.395	0.422	1.13 (0.97–1.32)	1.1E-01	1.17 (1.10–1.25)	2.69E-06	++
rs2107272	7	43,156,788	HECW1	C	T	0.342	0.296	0.325	0.301	0.85	3.6E-05	0.332	0.295	0.83 (0.71–0.98)	2.5E-02	0.85 (0.79–0.91)	3.04E-06	–
rs6846520	4	83,097,119	PLAC8	G	A	0.071	0.109	0.089	0.108	1.35	3.9E-06	0.085	0.094	1.14 (0.88–1.47)	3.3E-01	1.30 (1.16–1.46)	4.87E-06	++
rs12744678	1	204,967,707	NFASC	C	T	0.331	0.280	0.302	0.287	0.86	2.1E-04	0.319	0.273	0.80 (0.68–0.94)	5.6E-03	0.85 (0.79–0.91)	6.22E-06	–
rs4733780	8	130,284,009	ASAP1	T	C	0.454	0.423	0.446	0.414	0.86	3.2E-05	0.457	0.429	0.89 (0.77–1.03)	1.1E-01	0.86 (0.81–0.92)	9.94E-06	–
rs6852535	4	122,557,561	IL2/IL21	G	A	0.289	0.338	0.277	0.322	1.24	9.2E-08	0.296	0.283	0.93 (0.79–1.09)	3.8E-01	1.17 (1.09–1.26)	1.11E-05	+−
rs10509119	10	60,052,882	ANK3	T	C	0.131	0.110	0.133	0.112	0.80	4.1E-05	0.130	0.113	0.83 (0.67–1.04)	1.1E-01	0.81 (0.73–0.89)	1.29E-05	–
rs11902595	2	48,456,607	PPP1R21	T	G	0.146	0.180	0.159	0.180	1.22	8.3E-05	0.161	0.184	1.21 (0.99–1.47)	6.1E-02	1.21 (1.11–1.33)	1.41E-05	++
rs2142991	10	42,845,657	BMS1/ LOC101929445	T	C	0.139	0.173	0.139	0.169	1.30	4.9E-07	0.156	0.150	0.95 (0.77–1.16)	6.0E-01	1.22 (1.11–1.34)	2.13E-05	+−
rs406113	6	28,515,705	GPX6	T	G	0.340	0.297	0.343	0.311	0.86	6.9E-05	0.339	0.314	0.90 (0.78–1.06)	2.0E-01	0.87 (0.81–0.93)	3.31E-05	–
rs4814911	20	19,863,250	RIN2	G	A	0.225	0.190	0.212	0.193	0.84	7.7E-05	0.214	0.195	0.89 (0.75–1.07)	2.3E-01	0.85 (0.78–0.92)	4.40E-05	–
rs17199690	16	25,420,834	LOC105371146	G	A	0.172	0.137	0.160	0.136	0.80	1.3E-05	0.144	0.139	0.97 (0.79–1.21)	8.2E-01	0.83 (0.76–0.91)	4.41E-05	–
rs895606	9	85,388,753	NTRK2/ AGTPBP1	C	T	0.496	0.444	0.472	0.449	0.86	3.1E-05	0.475	0.465	0.96 (0.83–1.12)	6.2E-01	0.88 (0.82–0.94)	5.74E-05	–
rs11532787	7	119,661,732	ANKRD7/KCND2	G	A	0.138	0.112	0.121	0.109	0.81	1.9E-04	0.116	0.102	0.86 (0.68–1.08)	1.9E-01	0.82 (0.74–0.91)	9.73E-05	–
rs3017756	11	95,804,194	CEP57	C	T	0.482	0.426	0.438	0.429	0.86	6.6E-05	0.428	0.418	0.95 (0.82–1.10)	5.0E-01	0.88 (0.82–0.94)	9.96E-05	–
rs11028138	11	24,751,636	LUZP2	T	C	0.102	0.081	0.085	0.068	0.75	6.8E-06	0.085	0.089	1.08 (0.83–1.41)	5.7E-01	0.80 (0.71–0.90)	1.42E-04	−+
rs13025979	2	186,000,879	LOC107985783	C	T	0.099	0.074	0.098	0.077	0.75	3.9E-06	0.080	0.088	1.15 (0.87–1.51)	3.3E-01	0.80 (0.72–0.90)	1.60E-04	−+
rs10083119	12	125,824,612	TMEM132B/ LOC101927464	G	A	0.126	0.096	0.119	0.103	0.80	8.0E-05	0.112	0.106	0.95 (0.75–1.20)	6.6E-01	0.83 (0.75–0.91)	2.07E-04	–
rs4890490	18	44,857,303	SETBP1	C	T	0.436	0.385	0.413	0.402	0.88	9.0E-04	0.434	0.410	0.89 (0.77–1.03)	1.2E-01	0.89 (0.83–0.95)	2.76E-04	–
rs1433277	10	125,584,032	TEX36	G	T	0.431	0.472	0.433	0.466	1.17	2.1E-05	0.460	0.450	0.96 (0.83–1.12)	6.1E-01	1.13 (1.06–1.20)	3.24E-04	+−
rs4256646	9	73,182,919	ANXA1/ LOC101927281	T	C	0.458	0.509	0.466	0.490	1.16	9.6E-05	0.484	0.491	1.02 (0.88–1.18)	8.1E-01	1.13 (1.06–1.20)	3.33E-04	++
rs2075650	19	44,892,362	TOMM40	T	C	0.112	0.141	0.117	0.140	1.26	2.8E-05	0.144	0.138	0.96 (0.77–1.19)	6.9E-01	1.19 (1.08–1.32)	3.99E-04	+−
rs9783599	14	77,970,692	ADCK1 NRXN3	C	A	0.494	0.443	0.471	0.446	0.87	9.6E-05	0.469	0.475	1.05 (0.90–1.21)	5.4E-01	0.90 (0.84–0.96)	9.93E-04	−+
rs4742987	9	106,119,937	TMEM38B/ LOC100996590	T	C	0.393	0.359	0.389	0.364	0.86	6.8E-05	0.368	0.380	1.05 (0.90–1.22)	5.1E-01	0.89 (0.84–0.96)	1.08E-03	−+
rs1001408	12	66,898,508	GRIP1	A	G	0.119	0.086	0.103	0.095	0.81	5.1E-04	0.101	0.097	0.96 (0.76–1.23)	7.7E-01	0.84 (0.75–0.93)	1.15E-03	–
rs10197080	2	223,231,568	KCNE4/RN7SL807P	G	T	0.113	0.082	0.095	0.087	0.79	1.3E-04	0.100	0.104	1.08 (0.85–1.38)	5.2E-01	0.84 (0.75–0.94)	1.71E-03	−+
rs4768559	12	44,576,131	NELL2	T	G	0.262	0.209	0.224	0.214	0.85	2.1E-04	0.199	0.209	1.06 (0.89–1.28)	5.1E-01	0.89 (0.82–0.96)	1.77E-03	−+
rs217727	11	1,995,678	MRPL23/IGF2	G	A	0.218	0.178	0.189	0.177	0.85	3.5E-04	0.179	0.184	1.03 (0.85–1.24)	7.9E-01	0.88 (0.81–0.95)	2.02E-03	−+
rs7104402	11	43,019,438	LOC100507205/HNRNPKP3	C	T	0.323	0.281	0.302	0.290	0.87	6.2E-04	0.301	0.319	1.08 (0.92–1.27)	3.4E-01	0.91 (0.85–0.98)	8.58E-03	−+

Interestingly, our combined analysis identified two novel genome-wide significant associations, the first for *SLCO1A2* rs11568563 (OR = 0.67, *P* = 5.3E-10) and the second for *DUSP10* rs6687758 (OR = 0.8, *P* = 1.14E-8); both of these variants displayed associations with PSP in the previous GWAS that did not quite meet genome-wide significance. Importantly, associations for these two variants replicated in the independent patient-control series utilized in the current study (*SLCO1A2*: OR = 0.60, *P* = 0.0004; *DUSP10*: OR = 0.80, *P* = 0.017), where effect sizes and patient-control allele frequencies were very similar to those from stages 1 and 2 of the GWAS.

The top SNP in the *SLCO1A2* region (rs11568563), encodes a non-synonymous p.E172D mutation which may impact SLCO1A2 expression and/or function. To determine the association of *SLCO1A2* rs11568563 with abnormal gene expression of *SLCO1A2* or neighboring genes, we queried available gene expression databases for associated expression patterns and expanded this search to include all the markers in high LD with this variant (rs11568563 GWAS signal set, Table 3). The rs11568563 GWAS signal set was found to have 4 more intronic *SLCO1A2* variants (rs145667214, rs7966334, rs74651308 and rs79424089) and one intergenic variant close to the *SLCO1A2* 3’UTR (rs188509290) (Additional file 1: Figure S1A), all of which were found to have a modest non-significant effect on the regional brain expression of 17 genes (Additional file 2: Figure S1B). However, none of these 6 SNPs were found to have a significant eQTL effect, or significant regulatory functions on these genes. We did observe that five of these genes were found to be highly expressed in the brain, with three of these genes being mainly expressed in the brain (*SLCO1A2*, *C12 orf39* and *SLCO1C1*), and in regions affected in PSP (including the substantia nigra, caudate, putamen, and nucleus accumbens).

**Table 3:** GWAS signal set for rs 11,568,563

chr	GRCh38 location	r2	D’	SNP ID	SNP function annotation	Motifs	Regulome DB score
12	21,261,652	0.84	0.92	rs188509290	intergenic	Arid5a, Cdx, Cdx2, Dbx1, Foxa, Foxf1, Foxi1, Foxj2, Foxl1, HDAC2, Ncx, PLZF, Pou2f2, Pou3f4, TATA	6
12	21,304,500	1	1	rs11568563	SLCO1A2 missense	Ets, NF-kappaB, Rad21	6
12	21,312,146	1	1	rs145667214	SLCO1A2 intronic	–	ND
12	21,314,281	1	−1	rs7966334	SLCO1A2 intronic	BATF, Irf, LBP-9, SRF	ND
12	21,323,155	1	1	rs74651308	SLCO1A2 intronic	CEBPB, Evi-1, Hltf, Mef2, NF-E2, PLZF	ND
12	21,332,593	0.98	1	rs79424089	SLCO1A2 intronic	Nkx2	ND

A similar approach was taken to study the possible functional impact of rs6687758, the top SNP in our second newly identified genome-wide associated region. This variant is located in an ~ 223 Kb intergenic region between DUSP10 (~ 250 Kb upstream) and TRT-TGT2–1 (~ 473 Kb downstream) that contains 6 uncharacterized sequences and 3 pseudogenes. The rs6687758 variant is an eQTL for the expression of three large intergenic non-coding RNAs (lincRNAs): RP11-815 M8.1 (p 1.2E-15 in lung and 2.5E-5 in blood), RP11-400 N13.2 (3.3E-13 in lung) and LINC01655 (2.7E-8 in lung) but the expression of these sequences is negligible in the brain. The rs6687758 GWAS signal set included 50 variants (Table 4 and Additional file 2: Figure S2A) and none of them locate directly in well characterized, coding genes (Additional file 2: Figure S2B). This GWAS signal set was found to be associated with differential brain expression of eight genes (*c1orf140*, *LOC100287182*, *DUSP10*, *HHIPL2*, *MIA3*, *AIDA*, *C1orf58*, and *FAM177B*). Additionally, the effect of rs6687758 may be mediated by other LD variants.

**Table 4:** GWAS signal set for rs6687758

chr	GRCh38 location	r2	D’	SNP ID	SNP function annotation	Motifs	Regulome DB score
1	221,939,292	0.81	0.91	rs17011141	intergenic	DMRT1	5
1	221,940,306	0.81	0.91	rs17011146	intergenic	Barx2, Cdx2, Cdx2, Dbx2, Foxf1, Foxj1, Foxo, Hlx1, Hmx, Hoxa10, Hoxa9, Hoxb13, Hoxb9, Hoxc9, Hoxd8, Myc, Ncx, Nkx6–1, PLZF, Sox	6
1	221,940,827	0.81	0.91	rs72740077	intergenic	COMP1, PPAR, RXR::LXR, RXRA, RXRA	5
1	221,943,856	0.81	0.91	rs7520544	intergenic	Irf, Pax-5	ND
1	221,948,533	0.8	0.91	rs12123928	intergenic	Egr-1, Pax-2	6
1	221,952,186	0.81	0.91	rs12137323	intergenic	–	7
1	221,954,218	0.81	0.91	rs138207318	intergenic	Zic	ND
1	221,967,912	0.82	0.91	rs148737902	intergenic	–	ND
1	221,974,208	1	1	rs371061408	intronic RP11-815 M8.1 and RP11-400 N13.2	–	ND
1	221,974,503	0.89	0.97	rs12024555	intronic RP11-815 M8.1 and RP11-400 N13.2	EWSR1-FLI1, Zbtb3	7
1	221,974,877	0.89	0.96	rs149685934	intergenic	CACD, Pou1f1	6
1	221,975,001	0.911	1	rs12025420	intronic RP11-815 M8.1 and RP11-400 N13.2	–	6
1	221,975,210	0.884	1	rs12032598	intronic RP11-815 M8.1 and RP11-400 N13.2	–	5
1	221,975,457	0.94	0.98	rs12032653	intronic RP11-815 M8.1 and RP11-400 N13.2	LF-A1	5
1	221,975,480	0.94	0.98	rs12029332	intronic RP11-815 M8.1 and RP11-400 N13.2	BCL, NRSF	5
1	221,975,548	0.92	0.98	rs12025565	intronic RP11-815 M8.1 and RP11-400 N13.2	CAC-binding-protein, EWSR1-FLI1, Ets, Pax-3	6
1	221,975,629	0.88	0.98	rs12032715	intronic RP11-815 M8.1 and RP11-400 N13.2	Foxo, GR, Nanog, Sox	6
1	221,975,908	0.937	1	rs144092743	intronic RP11-815 M8.1 and RP11-400 N13.2	–	7
1	221,975,912	0.937	1	rs146528420	intronic RP11-815 M8.1 and RP11-400 N13.2	–	7
1	221,975,917	0.937	1	rs141093369	intronic RP11-815 M8.1 and RP11-400 N13.2	–	6
1	221,976,623	1	1	rs12026659	intronic RP11-815 M8.1 and RP11-400 N13.2	–	7
1	221,980,119	0.94	0.98	rs12129860	intronic RP11-400 N13.2	AP-2, Maf, Zbtb3	7
1	221,981,726	0.99	1	rs12033415	intronic RP11-400 N13.2	–	5
1	221,985,808	0.99	1	rs12140604	intergenic	Esr2, GATA, RORalpha1, Zbtb3	5
1	221,986,027 - 221,986,055	1	1	rs138253686	intergenic	–	7
1	221,988,647	1	1	rs6695584	intergenic	PPAR, RXRA	5
1	221,989,031	1	1	rs6691195	intergenic	–	ND
1	221,990,985	0.99	1	rs17011182	intergenic	Hlx1, Hoxa9, Nanog, Pou4f3, Sox	5
1	221,991,606	1	1	rs6687758	intergenic	Cdx2, Foxf1, Foxl1, Pou2f2, Pou3f3, TATA	5
1	221,995,092	0.97	1	rs12125383	intergenic	Nanog, Pou5f1, Pou5f1	4
1	221,996,081	0.92	0.99	rs34280100	intergenic	AP-1, AP-2, ATF3, BCL, BHLHE40, CCNT2, CHD2, E2F, EBF, Egr-1, Ets, GR, HEN1, Irf, Klf4, Klf7, MAZ, MAZR, MOVO-B, Myc, NRSF, Nrf1, PLAG1, Pax-4, Pou2f2, RREB-1, SP1, SRF, STAT, Sp4, TATA, UF1H3BETA, WT1, YY1, ZNF219, Zfp281, Zfp740, Znf143	5
1	222,002,851-222,002,852	0.939	1	rs141044286	intergenic	–	6
1	222,004,252-222,004,253	1	1	rs552801974	intergenic	–	NF
1	222,005,490-222,005,491	0.939	1	rs72221064	intergenic	–	6
1	222,006,808	0.94	0.99	rs17442058	intergenic	Hand1, Hoxa9, Hoxb13, Hoxb9, Hoxd10, Mef2, SP1, YY1, Zfp105	2b
1	222,009,847	0.92	0.98	rs12739936	intergenic	Zbtb3	7
1	222,013,781	0.92	0.98	rs11118926	intergenic	Egr-1, Ets, MZF1::1–4, Nrf-2, Nrf1, Pax-5, TATA	5
1	222,015,280	0.92	0.98	rs35805428	intergenic	Bcl6b, Hoxb6, STAT	7
1	222,016,251	0.92	0.98	rs17495159	intergenic	CEBPA, CEBPB	6
1	222,016,322	0.92	0.98	rs35212520	intergenic	YY1	6
1	222,016,779	0.92	0.98	rs12731064	intergenic	EBF, Elf5	7
1	222,018,163	0.92	0.98	rs17011200	intergenic	CDP, E2F, Evi-1, Smad3	5
1	222,018,592	0.92	0.98	rs66666316	intergenic	AP-1, Egr-1, Ets, Glis2, MAZR, MOVO-B, Myc, Pax-4, RREB-1, SP1, SRF, UF1H3BETA, YY1, ZNF219, Zfp281, Zfp740	6
1	222,019,622	0.92	0.98	rs17442323	intergenic	CTCF	7
1	222,021,538	0.92	0.98	rs12121134	intergenic	Myc	7
1	222,024,527	0.91	0.97	rs56170536	intergenic	NF-AT, SPIB, TATA, ZEB1, p300	5
1	222,025,330	0.91	0.97	rs12125368	intergenic	RORalpha1, RXRA	7
1	222,026,325	0.91	0.97	rs66732677	intergenic	HNF1, Pbx-1, RFX5	ND
1	222,034,659-222,034,660	0.911	1	rs71167281	intergenic	–	ND
1	222,045,419	0.84	0.96	rs12135286	intergenic	EBF, Nkx2, PPAR	5
1	222,046,411	0.85	0.96	rs35718308	intergenic	Mef2, ZBTB33	7

## Discussion

This study confirms the known association of loci at *MAPT*, *MOBP*, *EIF2AK3* and *STX6* with the risk of PSP and reveals novel associations with *SLCO1A2* and the intergenic rs6687758 SNP. Additional analysis were performed in the associated variants and their GWAS signal sets (variants in high LD with the GWAS variants) to gather functional evidence that could explain the observed association. We consider that the association of *SLCO1A2* rs11568563 with PSP is likely to be mediated by the non-synonymous p.E172D change that this variant induces. The p.E172D change affects a relatively conserved region (phastCons = 1, PhyloP = 4.143) of the 4th transmembrane domain of the organic anion-transporting polypeptide SLCO1A2, and the change is predicted to be probably damaging and deleterious by Polyphen and SIFT respectively [13]. Solute carrier organic anion transporters (SLCOs), also known as organic anion-transporting polypeptides (OATPs) facilitate the uptake of drugs in specific organs and therefore they influence absorption, distribution and elimination of drugs, xenobiotics, hormones and toxins. SLCO1A2 is the most important SLCO in the human brain, and the expression information collected from GTEx and Braineac confirms that it is highly expressed in the brain and in brain regions that are targets for tauopathy. Zhou et al. had shown previously that the rs11568563 minor allele is associated with low expression of *SLCO1A2* in the brain [6]. Specifically, the p.E172D change has been found to reduce the transport of known SLCO substrates [14] that is independent of protein expression or glycosylation but seems to be the result of altered SLCO1A2 cell surface trafficking and final localization to the plasma membrane.

Recently, a variation in *SLCO1A2* (rs73069071) has been associated with cortical Aβ deposition in AD-related cognitive impairment and temporal lobe atrophy and they proposed that this variant may be a modifier of Aβ deposition on AD-related neurodegeneration [15]. This variant is not in LD with rs11568563 but both SNPs are relatively close to each other in the *SLCO1A2* locus (139,057 bp distance): rs73069071 is located in the intron 2 and rs11568563 is located in the exon 7 (NM_134431)*.* Additionally, rs73069071 maps in an intronic region in the islet amyloid polypeptide, *IAPP,* which has been previously implicated in Alzheimer’s disease (AD) etiology [16]. The proximity of *IAPP* to *SLCO1A2* is due to *IAPP* genomic sequence being encoded in the complementary strand that spans from *SLCO1A2* intron 1 to intron 2. Studies suggest that *IAPP* expression is under the influence of rs11568563. Recent studies have shown that IAPP is an important regulator of apoptosis and autophagy [17], with both pathways linked to neurodegeneration. Since PSP is not associated with Aβ deposition but with tauopathy, it is possible that there is a more general role for SLCO1A2 in neurodegeneration and tau aggregation and that variation in *SLCO1A2* and/or *IAPP* underlie differential proteinopathies.

The effect of the intergenic rs6687758 variant is also unclear as the nearest gene, *DUSP10,* is located ~ 250 Kb away. In favor of its effect on *DUSP10*, a separate GWAS for colorectal cancer associated rs6687758 with *DUSP10* [18, 19]. rs6687758 is an eQTL for four lincRNAs (RP11-815 M8.1, RP11-400 N13.2, RP11-400 N13.3, and LINC01705) and it has been predicted to act as an enhancer. However, little is known about the function of these specific lincRNAs in the brain. Furthermore, the GWAS signal set associated with rs6687758 has 50 more variants mostly localized in a long intergenic region and with suggestive cis regulation of the neighboring genes *DUSP10, HHIPL2* and *FAM177B*. If involved, *DUSP10* may influence the accumulation of hyperphosphorylated tau, gliosis and synaptic/cognitive deficits due to the uncontrolled, hyperactivation of p38 and JNK kinases. However, a specific role of p38 and JNK in PSP will need to be elucidated since these MAPK pathways function in general cell signaling and are dysregulated in several neurodegenerative conditions.

Overall our strategy of expanding the PSP population, contrast it to age- and gender-matched controls and perform meta-analysis was successful in detecting genetic associations with PSP with a higher precision; however, this study cannot rule out that other variants that did not reach genome-wide significance are associated with PSP. Indeed, five of these variants had *p*-values lower than 1E-6 and replicated the direction of association observed in the GWAS (Table 2). Two of these SNPs (rs197971 and rs2107272) had even lower p-values than the GWAS (2.7E-6 vs 6.7E-6 and 3E-6 vs 3.6E-5 respectively) but further studies in larger case-control populations will be required to support the association of these non-genome-wide significant variants. Although all subjects were self-reported Caucasian, without genome-wide population control markers, and by focusing only on the 37 SNPs with the highest association in the previous PSP GWAS, our study cannot rule out population stratification influencing the observed results or explore the role of novel loci undetected by the original PSP GWAS.

## Conclusions

In conclusion, we have performed a meta-analysis adding a new PSP cohort to the previous GWAS population which confirmed that the top GWAS variants retain significant association with PSP and identified two novel associations with *SLCO1A2* and an intergenic rs6687758. Further studies are needed to understand the role of newly associated variants with PSP including the effect of *SLCO1A2* variation in BBB function in PSP and the *cis* and *trans* regulatory effects of GWAS variants on gene networks associated with tauopathy.

## Additional files

Additional file 1: **Figure S1.** *SLCO1A2* rs11568563 GWAS signal set. LD Manhattan plot for rs11568563 in the 1000G phase3:CEU as visualized using Ensembl (A). Genomic location of genes neighboring rs11568563 GWAS signal set as visualized in UCSC Genome Browser (B) with customized tracks from top to bottom: R2 plot for rs11568563, proxy variants in strong LD (r2 > 0.8) with rs11568563, USCS genes found to have differential brain expression and their associated GTEx RNA-seq gene expression (brain expression in yellow). (TIF 2794 kb)

Additional file 2: **Figure S2.** rs6687758 GWAS signal set. LD Manhattan plot for rs6687758 in the 1000G phase3:CEU as visualized using Ensembl (A). Genomic location of genes, predicted coding sequences and pseudogenes neighboring rs6687758 GWAS signal set as visualized in UCSC Genome Browser (B) with customized tracks from top to bottom: R2 plot for rs11568563, proxy variants in strong LD (r2 > 0.8) with rs6687758, USCS genes located in this region with the genes found to have differential brain expression highlighted in yellow and GTEx RNA-seq gene expression (brain expression in yellow). (TIF 2231 kb)

## Abbreviations

GWAS: Genomewide association study
PSP: Progressive supranuclear palsy
MAPT: Microtubule-associated protein tau
MOBP: Myelin-associated oligodendrocyte basic protein
EIF2AK3: Eukaryotic translation initiation factor 2 alpha kinase 3
STX6: Syntaxin 6
SLCO1A2: Solute carrier organic anion transporter family member 1A2
DUSP10: Dual specificity phosphatase 10
DWD: Dennis W. Dickson
SNP: Single nucleotide polymorphism
CEPH: The Centre d’Etude du polymorphism humain
LD: Linkage disequilibrium
eQTL: Expression quantitative locus
IRF4: Interferon regulatory factor 4
OR: Odds ratio
UTR: Untranslated region
lincRNA: Large intergenic non-coding RNAs
c1orf140: Chromosome 1 open reading frame 140
HHIPL2: Hedgehog interacting protein-like 2
MIA3: Melanoma inhibitory activity protein 3
AIDA: Axin interactor, dorsalization associated
C1orf58: Chromosome 1 open reading frame 58
FAM177B: Family with sequence similarity 177 member B
APOE: Apolipoprotein E
OATP1A2: Organic anion-transporting polypeptide 1A2
MAPK: Mitogen-activated protein kinase
JNK: Jun amino-terminal kinase
