# Supplementary figures and images for: Replication of progressive supranuclear palsy genome-wide association study identifies SLCO1A2 and DUSP10 as new susceptibility loci
Source: Mol Neurodegener. 2018 Jul 9;13:37. doi: 10.1186/s13024-018-0267-3 (PMC6038352; doi:10.1186/s13024-018-0267-3)

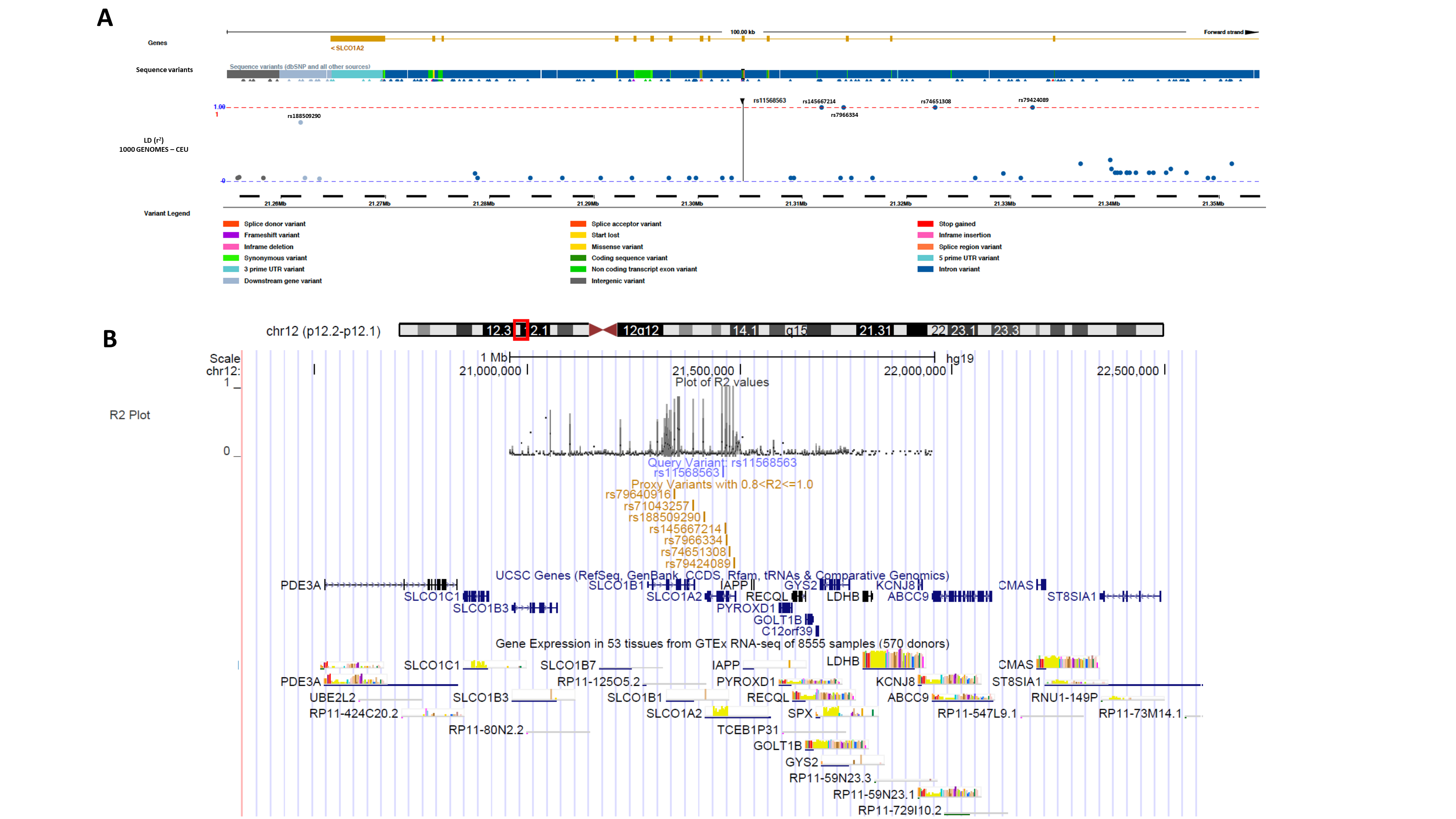

Supplement: Supplementary file 1 — Figure S1. SLCO1A2 rs11568563 GWAS signal set. LD Manhattan plot for rs11568563 in the 1000G phase3:CEU as visualized using Ensembl (A). Genomic location of genes neighboring rs11568563 GWAS signal set as visualized in UCSC Genome Browser (B) with customized tracks from top to bottom: R2 plot for rs11568563, proxy variants in strong LD (r2 > 0.8) with rs11568563, USCS genes found to have differential brain expression and their associated GTEx RNA-seq gene expression (brain expression in yellow). (TIF 2794 kb) [file 13024_2018_267_MOESM1_ESM.tif]

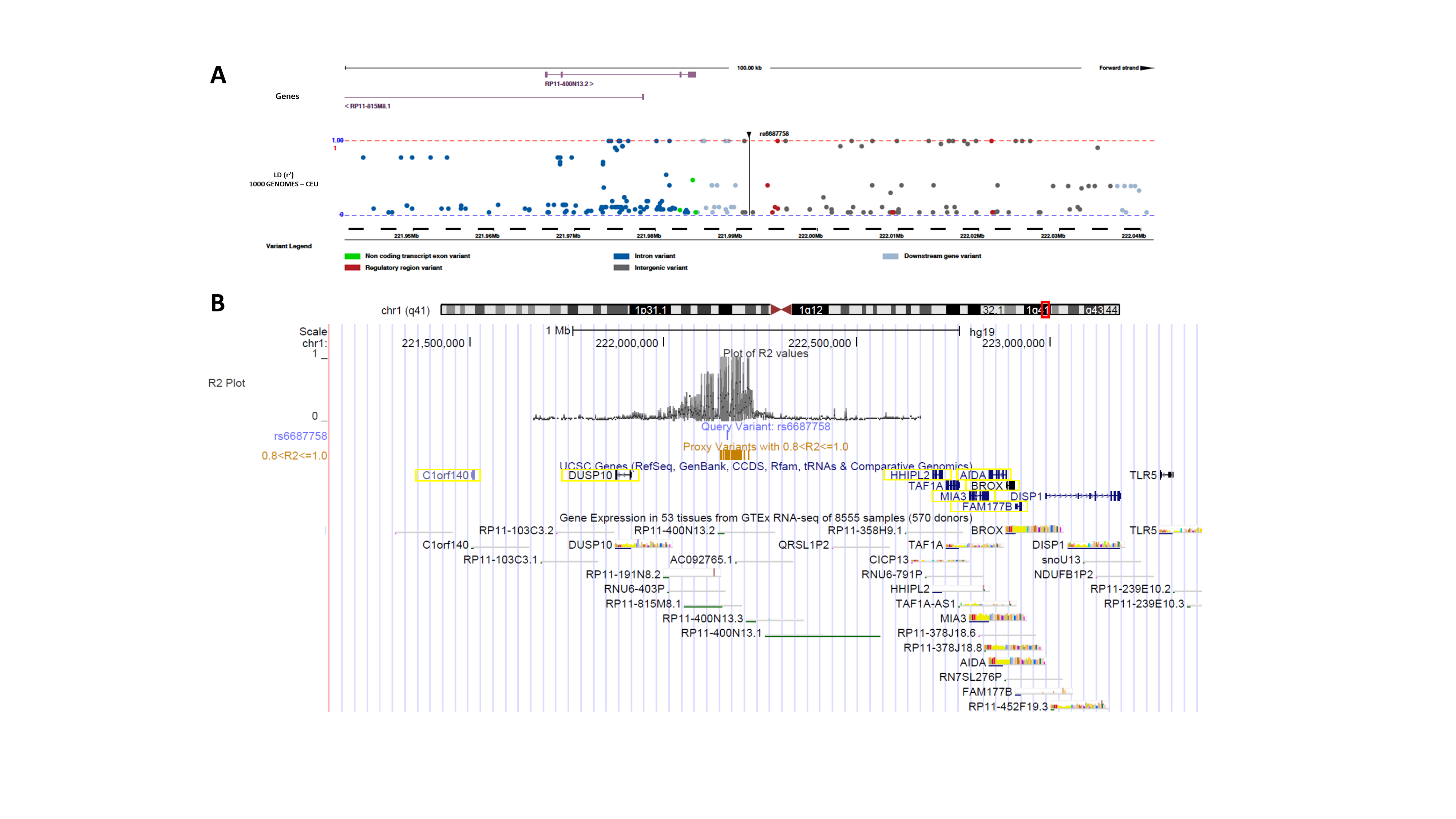

Supplement: Supplementary file 2 — Figure S2. rs6687758 GWAS signal set. LD Manhattan plot for rs6687758 in the 1000G phase3:CEU as visualized using Ensembl (A). Genomic location of genes, predicted coding sequences and pseudogenes neighboring rs6687758 GWAS signal set as visualized in UCSC Genome Browser (B) with customized tracks from top to bottom: R2 plot for rs11568563, proxy variants in strong LD (r2 > 0.8) with rs6687758, USCS genes located in this region with the genes found to have differential brain expression highlighted in yellow and GTEx RNA-seq gene expression (brain expression in yellow). (TIF 2231 kb) [file 13024_2018_267_MOESM2_ESM.tif]
